# The Impact of IPTG Induction on Plasmid Stability and Heterologous Protein Expression by *Escherichia coli* Biofilms

**DOI:** 10.3390/ijms21020576

**Published:** 2020-01-16

**Authors:** Luciana Gomes, Gabriel Monteiro, Filipe Mergulhão

**Affiliations:** 1 LEPABE—Department of Chemical Engineering, Faculty of Engineering, University of Porto, 4200-465 Porto, Portugal; luciana.gomes@fe.up.pt; 2 iBB—Institute for Bioengineering and Biosciences, Department of Bioengineering, Instituto Superior Técnico, Universidade de Lisboa, 1049-001 Lisboa, Portugal; gabmonteiro@tecnico.ulisboa.pt

**Keywords:** biofilm, *Escherichia coli*, heterologous protein expression, plasmid stability, plasmid copy number, IPTG

## Abstract

This work assesses the effect of chemical induction with isopropyl β-D-1-thiogalactopyranoside (IPTG) on the expression of enhanced green fluorescent protein (eGFP) by planktonic and biofilm cells of *Escherichia coli* JM109(DE3) transformed with a plasmid containing a T7 promoter. It was shown that induction negatively affected the growth and viability of planktonic cultures, and eGFP production did not increase. Heterologous protein production was not limited by gene dosage or by transcriptional activity. Results suggest that plasmid maintenance at high copy number imposes a metabolic burden that precludes high level expression of the heterologous protein. In biofilm cells, the inducer avoided the overall decrease in the amount of expressed eGFP, although this was not correlated with the gene dosage. Higher specific production levels were always attained with biofilm cells and it seems that while induction of biofilm cells shifts their metabolism towards the maintenance of heterologous protein concentration, in planktonic cells the cellular resources are directed towards plasmid replication and growth.

## 1. Introduction

*Escherichia coli* remains at the forefront of the expression systems used for the production of many recombinant proteins [1], despite the fact that it is unable to perform some post-translational modifications like glycosylation [1] and shows limited secretion capacity [2,3]. In fact, among marketed biopharmaceuticals for antitumoral therapies, 69% are produced in *E. coli* against only 26% produced in mammalian cells [4].

Successful recombinant protein production in *E. coli* is a combination of many good decisions involving the choice of the most appropriate strain, expression vector, cultivation and purification strategies [5]. Plasmids are the most commonly used vectors for the expression of recombinant proteins in *E. coli*. Their design is crucial in order to maintain an equilibrium between the transcriptional and translational machinery of the host cell so that the deleterious effects of heterologous protein production can be managed [2,5]. A plasmid contains many elements that are essential for its use as an expression vector, but some of the most important are the origin of replication and the promoter. The origin of replication controls the plasmid copy number (PCN) [1] and although it is often assumed that a high gene dosage is favorable for high level heterologous protein production, this is not always the case [5]. It has been shown that a high PCN may impose a metabolic burden that decreases the bacterial growth rate and originates plasmid instability, thus reducing the number of bacteria that are capable of high level protein synthesis [6,7,8].

Interestingly, plasmid stability is affected by biofilm formation. In a first study, Davies and Geesey [9] found that the PCN was approximately 1.5-fold higher in *Pseudomonas aeruginosa* biofilms than in planktonic cells. More recently, Cook and Dunny [10] showed that four non-conjugative plasmids had increased PCN (1.6- to two-fold) and copy-number heterogeneity in *Enterococcus faecalis* biofilm cells when compared to planktonic cells, and this increased PCN was correlated with the increased expression of plasmid-borne resistance genes. Additionally, higher PCN values were found in *E. coli* cells growing as biofilms comparatively to the suspended cultures: 400–500 versus 200–300 copies per cell in a chemostat (planktonic growth) [11] and 60 versus 40 copies per cell in planktonic cultures [12], both studies performed in the presence of antibiotic pressure. The potential of *E. coli* biofilm cells to retain high PCNs can be explained by the slower growth of sessile cells compared to their planktonic counterparts [13], leading to fewer divisions and correspondingly less plasmid segregation. Conversely, it has also been demonstrated that, for some plasmids, plasmid loss is more significant in biofilm populations [14,15] and that this can be affected by the age of the biofilm [16].

In this work, the pET system was used for the expression of a heterologous model protein, the enhanced green fluorescent protein (eGFP). This family of vectors contains a pMB1 origin of replication (medium-copy number replicon) and uses the T7 promoter for gene transcription. When the gene is under the control of the lac operator, isopropyl β-D-1-thiogalactopyranoside (IPTG) is usually added to induce protein expression [1]. This system is one of the most widely used expression systems in *E. coli* mainly due to its very high expression levels as the target protein can represent up to 50% of the total cell protein [1]. Even in the absence of IPTG, there is often a basal level expression of T7 RNA polymerase from the *lacUV5* promoter in λDE3 lysogens, leading to some basal or leaky expression of heterologous genes placed under the T7 promoter. We recently demonstrated that the non-induced eGFP expression from biofilm cells was 30-fold higher than in the planktonic state without any optimization of cultivation parameters [17]. The aim of the present work was to evaluate the effect of IPTG induction on heterologous protein production by biofilm cells and on plasmid stability.

## 2. Results

### 2.1. Effect of IPTG Induction on Planktonic and Biofilm Growth

**Figure 1:**
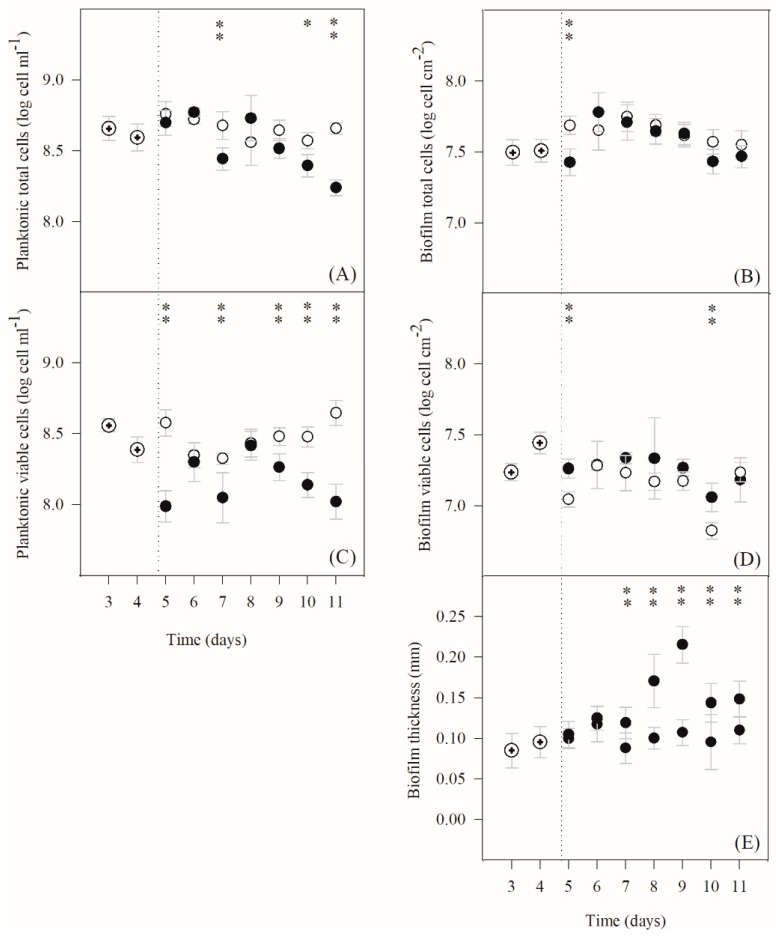
Time-course of planktonic and biofilm parameters: (**A**) planktonic total cells, (**B**) biofilm total cells, (**C**) planktonic viable cells, (**D**) biofilm viable cells, (**E**) biofilm thickness. Induced (⬤) and non-induced (◯) LB culture. The dotted lines indicate the day on which the culture was induced with 2 mM isopropyl β-D-1-thiogalactopyranoside (IPTG). The biofilm and planktonic samples corresponding to day 5 were collected 6 h after IPTG addition. Results are an average of three independent experiments for each condition, except in days 3 and 4 that result from an average of six independent experiments (three experiments for induced conditions and three experiments for non-induced conditions (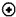)). The averages ± SDs (indicated as bars) are illustrated. Statistical analysis corresponding to each time point is represented with 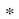 for a confidence level greater than 90% (*p* < 0.1) and with 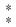 for a confidence level greater than 95% (*p* < 0.05).

The effect of IPTG induction on the dynamics of planktonic and biofilm growth is presented in Figure 1. The growth of non-induced and induced planktonic cells was compared by determining the number of total (Figure 1A) and viable cells (Figure 1C). Planktonic total cell concentration (Figure 1A) was almost constant throughout the experiment in the non-induced state, but a decrease was observed for the induced culture particularly towards the end of the experiment. Similar behavior was observed for the number of viable cells (Figure 1C), and statistically significant differences were found in the majority of experimental points (*p* < 0.05). Indeed, from day 5 onwards the values for the induced cells remained mostly lower (49%) than those determined in the non-induced culture. It is important to note that the IPTG decreased in the medium over time, but it was present until the end of the experiment at concentrations above 0.24 mM (see Supplementary Material, Figure S1).

The IPTG transport from the bulk medium to the top layer of the biofilm was also estimated. A maximum molar flux of 4.7 nmol·s^−1^ was obtained by estimation of the external mass transfer coefficient (Equation (8)) under the operational conditions. The impact of IPTG induction on biofilm development was also assessed by determining the number of total (Figure 1B) and viable biofilm cells (Figure 1D) and the biofilm thickness (Figure 1E). The total cell and viable counts were very similar throughout the experiment and did not change significantly after exposure to IPTG. Nevertheless, the biofilm thickness was significantly higher for the induced biofilms from day 7 until the end of the experiment (*p* < 0.05, Figure 1E).

The composition of extracellular polymeric substances (EPS) of 7-day-old biofilms was analyzed (Table 1), and no significant differences were observed with IPTG induction as the dry mass, cell density and also the protein and polysaccharide levels of the matrix were similar (*p* > 0.05).

### 2.2. Determination of eGFP, PCN, mRNA and Total RNA

**Figure 2:**
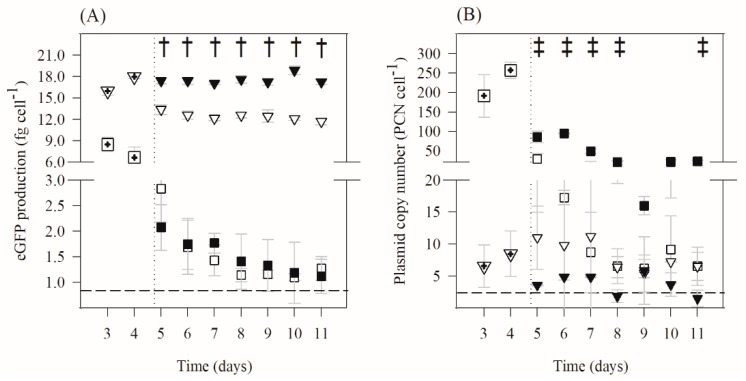
Time-course of (**A**) eGFP production and (**B**) plasmid copy number for planktonic and biofilm cells. Induced (▼) and non-induced (▽) biofilm cells; induced (■) and non-induced (☐) planktonic cells. The vertical dotted lines indicate the day on which the culture was induced with 2 mM IPTG, and the horizontal dashed lines correspond to detection limits of the methods. The biofilm and planktonic samples corresponding to day 5 were collected 6 h after IPTG addition. Results are an average of three independent experiments for each condition, except in days 3 and 4 that result from an average of six independent experiments (three experiments for induced conditions and three experiments for non-induced conditions; biofilm (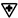) and planktonic (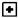) cells). The averages ± SDs (indicated as bars) are illustrated. Statistical analysis for a confidence level greater than 95% (*p* < 0.05) is pointed as † when induced biofilm cells are different from the non-induced biofilm cells and as ‡ when induced planktonic cells are different from the non-induced planktonic cells.

The variation of specific eGFP production and plasmid copy number during planktonic and biofilm growth was monitored for non-inducing and inducing conditions (Figure 2). Comparing expression in planktonic and sessile cells (Figure 2A), it is clear that the biofilm state increased heterologous protein production (six- and nine-fold for non-induced and induced cultures, respectively). Specific eGFP production from planktonic cells decreased (approximately 86%) between days 3 and 11 in both induced and non-induced states. Unexpectedly, the eGFP levels in induced planktonic cells were similar to the leaky expression from the non-induced cells. The eGFP production from biofilm cells remained approximately constant for the induced culture (pre-induction levels were maintained at around 17 fg·cell^−1^), whereas a decrease in production (of about 31%) was observed on the non-induced cells. Thus, it seems that IPTG induction had a more pronounced effect on biofilm than in planktonic cells.

The PCN (Figure 2B) was higher in planktonic than in sessile cells (on average seven- and 19-fold higher for non-induced and induced cultures, respectively, during the experimental time), showing that until day 3 the adhered cells lost most of the plasmid content. After day 5, when one of the cultures was induced, both induced and non-induced planktonic cells had their PCN reduced. This reduction was more pronounced in the non-induced culture (around 89%) than in the induced condition (approximately 66%). In biofilms, these reductions were smaller, since the PCN reduced about 38% in the induced cultures and there was no loss of plasmid in the non-induced cultures.

**Figure 3:**
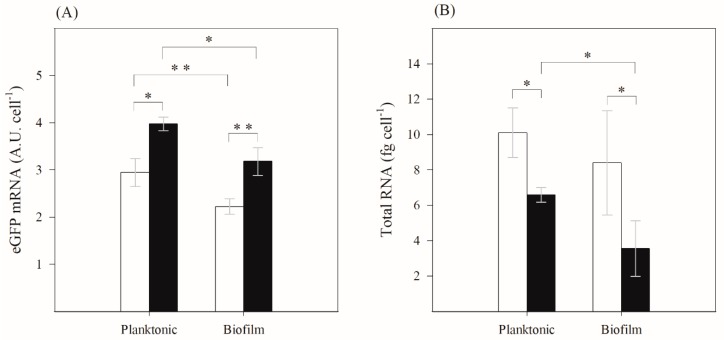
(**A**) Quantification of eGFP gene transcription and (**B**) total RNA concentration for planktonic and biofilm cells on day 7. Induced (■) and non-induced (☐) cells. The averages ± SDs (indicated as bars) for two independent experiments for each condition (considering the same quantity of cells) are illustrated. Statistical analysis corresponding to each time point is represented by 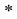 for a confidence level greater than 90% (*p* < 0.1) and by 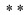 for a confidence level greater than 95% (*p* < 0.05).

The levels of mRNA and total RNA for day 7 (steady-state conditions) are presented in Figure 3. Higher transcription of the eGFP gene occurred in planktonic cells when compared to biofilms (average *p* < 0.1), independently of the presence of IPTG (Figure 3A). Furthermore, transcription was more intense upon induction in both planktonic and biofilm environments (average *p* < 0.05) (Figure 3A). Observing the total RNA levels (Figure 3B), higher values were obtained for planktonic cells (*p* < 0.1), and total RNA levels in both types of cells decreased after induction.

## 3. Discussion

The flow cell system used in this work can be considered as a chemostat with an irregular geometry [18,19]. For planktonic cells, under steady-state conditions, the dilution rate is equal to the specific growth rate of bacteria. A constant cellular concentration was reached for the non-induced planktonic culture, and therefore the specific *E. coli* growth rate was 0.015 h^−1^. Specific growth rates in batch culture were determined for this strain, and values around 0.7 h^−1^ were obtained, indicating that the dilution rate used in these experiments was much lower than the critical dilution rate for non-induced conditions. This low growth rate should be taken into consideration when analyzing our results as cells are in different physiological conditions when compared with the ones in most of the current publications regarding heterologous protein expression [11,20,21,22]. Since the planktonic cell concentration decreased for the induced culture, mostly towards the end of the experiment, the growth rate of these cells was below 0.015 h^−1^. Taking into account that the non-induced culture can grow at this temperature and in this growth medium at specific growth rates that far exceed this value (as determined in batch cultivation), it is plausible to assume that IPTG induction affected the metabolism of planktonic cells. A reduction in cell growth rates upon induction had already been demonstrated for plasmid-bearing cells [23,24,25]. It has been proposed that cell growth arrest is caused by the metabolic stress that an expression vector imposes upon induction, including sequestering of cellular resources for transcription and translation of the target gene. In the present study, since the amount of eGFP produced by induced and non-induced planktonic cells was similar, the reduction of cell density in the induced culture was possibly a consequence of the metabolic drain of biosynthetic precursors, energy and other cellular components for plasmid replication and transcription. IPTG induction had a negative effect not only on growth but also on cellular viability of *E. coli* suspended cultures. Although IPTG was added in a single pulse, it was estimated that the inducer was present in the bulk medium at high concentrations (above 0.24 mM) until the end of the experiment. This long term exposure may explain the deleterious effects on the growth and viability of planktonic cells.

IPTG addition had different effects on planktonic and biofilm cells. As far as biofilm development is concerned, there were no major observed effects on the biofilm cell number, viability or even EPS composition. It was therefore important to estimate if the IPTG that was added as a pulse to the bulk medium would reach the biofilm so that biofilm cells could be induced. A crude estimation of the external mass transfer coefficient allowed us to estimate that a molar flux of 4.7 nmol s^−1^ can reach the surface of the biofilm. Although this does not guarantee that the inducer reaches all the biofilm cells, previous studies have shown that this molecule readily penetrates biofilms, and it has been estimated that it can take up to 7 min for IPTG to cross a 300 µm-thick biofilm [26,27]. Heterologous protein expression levels in our system were maintained from day 5 onwards. On that day, biofilm thickness was around 100 µm, and therefore it is very likely that IPTG was able to cross the biofilm effectively during the timescale of the experiment. Additionally, since IPTG is not hydrolyzed by the cells [28], its concentration in the growth medium can be estimated by the simple mass balance performed in this work, which suggested that this compound was present in the culture medium until the end of the experiment.

Regarding biofilm formation, the non-accumulation of bacterial cells after induction corroborates the results obtained by Huang et al. [29] using *E. coli* DH5α(pTKW106) cultures exposed to the highest IPTG concentration tested (0.51 mM). However, the biofilm thickness was higher for the induced biofilms from day 7 onwards. Since the EPS content of induced and non-induced biofilms was similar, it is possible that the increase of thickness was an architectural change of the biofilm itself in an attempt to adapt to the introduction of IPTG in the culture medium. In a previous work, Teodósio et al. [30] showed that *E. coli* JM109(DE3) biofilms adjust their architecture once they reach a certain thickness, possibly to increase internal nutrient transfer to the inner layers of the biofilm.

By comparing the eGFP concentrations in planktonic and biofilm cells, it is possible to conclude that the inducer had an instantaneous effect on sessile cells, preventing the overall decrease in the amount of expressed eGFP. A fast IPTG response was also obtained by Stewart [27] and Lenz et al. [31], who reported the appearance of a green fluorescent band in *P. aeruginosa* biofilms after only 4 h of induction, whether IPTG was applied from the bottom of colony biofilms or from the liquid medium in drip-flow cultivated biofilms. Other reports on *E. coli* biofilms [29,32] confirmed the variation in recombinant protein concentration in the first hours of induction. In our work, a lower amount of eGFP mRNA was detected after induction in biofilm cells compared to planktonic cells. However, since lower levels of total RNA were also measured in biofilm cells and these are essentially ribosomal RNA (rRNA) [33], it is possible that the biofilm was metabolically less active, and therefore more cellular resources were available to produce the heterologous protein. It is interesting to observe that upon induction, the total RNA levels of both planktonic and biofilm cells decrease. This effect was previously reported by Dong et al. [34] and it can be an indication that the overall protein translation activity is decreased in induced cells. In our system, a relatively constant biofilm cell number was observed after induction. Thus, biofilm growth rate was similar to biofilm detachment rate. If cells are growing more slowly in biofilms [13], the production of housekeeping proteins also decreases, and consequently more cellular resources will become available for heterologous protein production.

Contrary to what was expected, heterologous protein production from planktonic cells did not benefit from IPTG induction, since expression levels were similar to the leaky expression from the non-induced culture. We showed that IPTG was present in the culture medium until the end of the experiment, and it has been shown that a six-fold lower concentration than what was estimated may be enough for full induction [28]. Despite this fact, it has also been reported that addition of IPTG in the culture medium may be detrimental in the expression of some types of recombinant proteins, particularly when LB medium is used [35]. It has also been suggested that IPTG addition can be deleterious in the expression of recombinant proteins when using high-copy plasmids [36]. The pET plasmid contains a pMB1 origin of replication and is considered a medium-copy number plasmid with an expected PCN between 15 and 60 [1]. Although we have shown that it can reach a PCN of about 200 copies, its steady state values are closer to 50 (for the induced planktonic culture) or around 10 or below for biofilm cells and non-induced planktonic cultures. It is interesting to observe that the most beneficial effects of induction were observed in the cells that contain a lower PCN.

Although a high concentration of IPTG was available in the growth medium, both induced and non-induced planktonic cells presented the same specific eGFP concentration. We have already concluded that heterologous protein production from induced planktonic cells is not limited by inducer concentration, gene dosage or transcriptional activity. The translational capacity of induced planktonic cells is probably higher than in induced biofilm cells as total RNA levels are also higher. Thus, the low level eGFP production from induced planktonic cells may be due to the high metabolic burden associated with plasmid replication, since PCN in induced planktonic cultures is on average five-fold higher than in non-induced cells. Plasmid replication probably causes a high metabolic drain not only in terms of the requirement of nucleotides but also in the expression of the antibiotic resistance gene [7]. Thus, for producing the same amount of eGFP, the higher levels of total RNA (and therefore ribosomes) in planktonic non-induced cells may compensate for the lower gene dosage effect of these cells when compared to planktonic induced cells. The absence of an increase in the eGFP concentration on induced cells may be related to the leakage of recombinant protein into the extracellular medium caused by the loss of membrane integrity, as previously reported by Lowder et al. [37]. This phenomenon of cell lysis is supported by the strong decline in the number of viable cells in suspended cultures immediately after IPTG addition.

It is well known that plasmid stability is a key issue in recombinant protein production [5]. There is a considerable amount of information available on the plasmid stability in *E. coli* batch cultures (e.g., [6,7,8]), but little is known about the influence of biofilm development on the plasmid copy number and recombinant protein expression. In this work, higher specific eGFP concentrations were detected in biofilm cells, where a smaller number of copies of plasmid pFM23 was found compared to planktonic cells. Even within biofilms cells, a higher PCN was obtained in non-induced cells, whereas higher eGFP production was achieved with induction. Thus, the gene dosage was not controlling the production of eGFP in both types of cells. The same phenomenon was observed by Bhattacharya and Dubey [38] in *E. coli* suspensions where induced cultures had 17% more foreign protein, even though they only presented a PCN of 65 against 75 of non-induced cultures. Similarly to our results, the increased metabolic burden derived from the higher recombinant protein production in biofilm cells may have itself contributed to PCN decrease, as observed by Ricci and Hernández [39] for batch cultures.

From the PCN results, it can be established that the biofilm mode of growth had a beneficial effect on plasmid retention in a dividing population. This agrees with the results obtained in previous studies [9,11,12] and can be explained by the fact that cells in biofilms tend to grow more slowly than their planktonic counterparts [13], leading to fewer cell divisions and correspondingly less plasmid segregation.

## 4. Materials and Methods 

### 4.1. Bacterial Strain and Culture Medium

*E. coli* JM109(DE3) harbouring the plasmid pFM23 was used in this work for the cytoplasmic production of eGFP [40]. The strain was grown in Lysogeny broth (LB-Miller; Sigma, St. Louis, MO, USA) supplemented with 20 µg mL^−1^ kanamycin (Eurobio, Courtaboeuf, France). In a previous work, the change in the nutritional composition of growth medium from a diluted medium [17] to LB led to a two-fold increase in the eGFP production of biofilm cells [41]. Furthermore, LB containing 20 µg mL^−1^ kanamycin was the most advantageous medium to obtain the highest specific production in the sessile state [41].

### 4.2. Biofilm Formation System

A flow cell system [42] was operated under turbulent conditions (Re = 4600) by recirculating the bacterial suspension at 30 °C during 11 days. The biofilms were formed in polyvinyl chloride (PVC) slides (2 × 1 cm) fixed to the removable coupons of the flow cell (a semi-circular Perspex duct with 3.0 cm diameter and 1.2 m length). The recirculating tank of 1 L was continuously fed with 0.025 L h^−1^ of LB.

Following an initial screening to find the optimal inducer concentration and induction point, the flow cell was operated for 5 days, and induction was performed by adding a single pulse of IPTG (BIORON GmbH, Ludwigshafen, Germany) in order to obtain a final concentration of 2 mM in the medium. The biofilm and planktonic samples corresponding to day 5 were collected 6 h after IPTG addition.

The system can be considered as a well-mixed chemostat with irregular geometry [19], thus the concentration of any substance in the effluent stream is identical to the concentration inside the system (Equation (1)) [43]. A mass balance for the IPTG that was introduced as a pulse at time *t* = 0 into the recirculating tank yields for *t* > 0.

(1) In−Out=Accumulation

(2) $$0-FC=V\frac{dC}{dt}$$

*C* in Equation (2) is the concentration of IPTG either in the effluent or inside the system. *F* is the volumetric flow rate, and *V* is the volume of the flow cell system. Separating the variables and integrating with *C* = *C*_0_ at *t* = 0 yields Equation (3).

(3) $$C\left(t\right)=C_{0}e^{-t/\tau }$$

where *τ* is the average residence time given by Equation (4).

(4) $$\tau =\frac{V}{F}$$

Equation (3) enables the calculation of the IPTG concentration inside the reactor at any given time *t*, which is represented for the flow cell system operating for a further 6 days after IPTG addition (see Supplementary Material, Figure S1). The dilution rate (*D*) of the chemostat is given by Equation (5).

(5) $$D=\frac{F}{V}$$

### 4.3. Estimation of IPTG Transfer Rate

The rate of IPTG transport from the bulk liquid to the slow-moving fluid adjacent to the biofilm was estimated by the external mass transfer coefficient (*K_m_*).

The Sherwood number (*Sh*) for fully developed turbulent flow conditions was determined using Equation (6) for *Re* numbers ranging from 2100 to 35,000 and Schmidt numbers (*Sc*) ranging from 0.6 to 3000 [44].

(6) Sh=0.023 Re0.83Sc13

The external mass transfer coefficient was estimated by Equation (7) taking into account the *Sh* number previously determined.

(7) $$K_{m}=\frac{Sh\;D_{aq}}{d}$$

where *D_aq_* is the diffusion coefficient of IPTG in water (8.7 × 10^−10^ m^2^·s^−1^ at 30 °C) [27], and *d* is the hydraulic diameter.

Immediately after IPTG addition, the concentration at the top of the biofilm is approximately zero, and therefore the mass transfer rate ($\dot{n}$) can be estimated by

(8) $$\dot{n}=K_{m}AC$$

where *A* is the coupon area.

### 4.4. Biofilm and Planktonic Analysis

One coupon was taken from the flow cell on each test day, and the biofilm thickness was immediately measured using an electronic digital micrometer [30]. Then, the biofilm was resuspended and homogenized in 25 mL of 8.5 g L^−1^ NaCl solution for assessment of total and viable cells and for quantification of eGFP and plasmid content. The Live/Dead^®^ BacLight™ bacterial viability kit (Syto9/propidium iodide; Invitrogen Life Technologies, Alfagene, Carcavelos, Portugal) was used to quantify the viability of bacterial populations as a function of the membrane integrity of the cell [45]. Results of total (viable plus non-viable) and viable cell counts were expressed as log cell·cm^−2^.

For planktonic cells, total and viable cell numbers were determined using the same method as for biofilms, and the results were presented as log cell·mL^−1^.

### 4.5. Quantification of EPS

After 7 days of biofilm growth, the number of proteins and polysaccharides in EPS was quantified. Extraction of matrix proteins and polysaccharides from sessile cells was carried out using a Dowex resin (50 × 8, Na^+^ form, 20–50 mesh; Fluka Chemika, Buchs, Switzerland) as indicated in Gomes et al. [46]. Protein concentration in EPS (matrix constituents) was quantified using the BCA^TM^ Protein Assay Kit (Thermo Fisher Scientific, Waltham, MA, USA) using bovine serum albumin as standard. The polysaccharide content was measured by using the phenol-sulfuric acid assay described by DuBois et al. [47] using glucose as standard. Negative controls for both methods were also prepared. The final values resulted from three independent experiments in the flow cell system and were calculated taking into account the biofilm dry weight determined as indicated by Simões et al. [48].

### 4.6. Quantification of eGFP Production

The eGFP expression in both planktonic and biofilm cells was analyzed as described by Gomes and Mergulhão [17]. A volume of planktonic and biofilm suspension corresponding to an equivalent OD_610 nm_ of 1 (equivalent to a cellular density of 7.6 × 10^8^ cells·mL^−1^ [30]) was used to harvest the cells by centrifugation. The 200 μL of Buffer I (50 mM Na_2_HPO_4_, 300 mM NaCl, pH 8) was added to resuspend each pellet, and a 96-well microtiter plate reader (SpectraMax M2E; Molecular Devices, Inc., Wokingham, UK) was used to measure the recombinant protein fluorescence (488 nm excitation; 507 nm emission). Calibration curves of fluorescence intensity and its corresponding eGFP concentration were constructed, and the expression levels of recombinant protein were shown as fg cell^−1^ [40]. The remaining volume of planktonic and biofilm samples was centrifuged, and the pellets were immediately frozen for subsequent quantification of PCN and evaluation of eGFP gene transcription.

### 4.7. Quantification of PCN

#### 4.7.1. Preparation of Plasmid DNA (pDNA) Standards

*E. coli* JM109(DE3) cultures for pDNA preparation were grown from a single colony picked from a freshly streaked LB agar plate supplemented with 20 µg mL^−1^ kanamycin. The plasmid purification was made using the High Pure Plasmid Isolation Kit protocol (Roche Diagnostics GmbH, Mannheim, Germany). The plasmid concentration was determined with a NanoDrop Spectrophotometer (NanoVue^TM^ Plus; GE Healthcare, Freiburg, Germany), and its quality was assessed by gel electrophoresis (agarose 1% in TAE buffer 1×). Serial dilutions of plasmid (1, 10, 100, 1000 and 10,000 pg per capillary) were conducted in triplicates to establish the standard curve.

#### 4.7.2. Real-Time PCR (RT-PCR)

The quantification of plasmid copy number in planktonic and biofilm samples was carried out in the Roche LightCycler^TM^ detection system using the FastStart DNA Master SYBR Green I Kit^TM^ (Roche Diagnostics GmbH, Penzberg, Germany) [49] by amplification of a 108 bp fragment of the eGFP gene (forward primer: 5′-TCGAGCTGGACGGCGACGTAAA-3′; reverse primer: 5′-TGCCGGTGGTGCAGATGAAC-3′).

Reaction mixtures (20 µL) had 2 µL of the 10× SYBR Green mixture, 1 µL of each primer (0.5 µM final concentration), 1.6 µL of MgCl_2_ solution (3 mM final concentration), 2 µL of sample prepared as described below and 12.4 µL of PCR-grade deionized water. Reaction mixtures were kept at 95 °C for 10 min to activate the FastStart DNA polymerase, and then the amplification step was started (30 cycles of 15 s at 95 °C, 10 s at 55 °C and 14 s at 72 °C). After the final cycle, reactions were incubated at 70 °C for 30 s and denatured with a temperature gradient from 70 to 95 °C at 0.1 °C·s^−1^.

Plasmid DNA samples for the construction of a calibration curve were prepared by spiking 2 µL of purified pDNA standards with non-transformed *E. coli* JM109(DE3) cells.

Appropriate dilutions of the frozen pellets were made with MilliQ water in order to maintain the same number of total cells per reaction (3 × 10^5^ cells). Determination of pDNA concentration in planktonic and biofilm cells was done for triplicate sets by mixing 2 µL of suspension with 3 × 10^5^ of cells, 2 µL of PCR grade water and the other PCR reagents as described above.

The calibration curve was established according to the method of Lee et al. [50]. Briefly, it includes the plot of the cycle threshold (C_T_) values versus the log concentration of the plasmid DNA standard. For the unknown pDNA samples, the plasmid concentration was obtained by interpolating its C_T_ value against the calibration curve. The corresponding PCN was then calculated using the Equation (9) [51]:

(9) $$\mathrm{PCN}=\frac{6.02\times 10^{23}\;\left({\mathrm{copy}\;\mathrm{mol}}^{-1}\right)\times \mathrm{DNA}\;\mathrm{amount}\;\left(g\right)}{\mathrm{DNA}\;\mathrm{length}\;\left(\mathrm{bp}\right)\times 660\;\left({g\;\mathrm{mol}}^{-1}{\mathrm{bp}}^{-1}\right)}$$

knowing that the size of plasmid pFM23 is 6053 bp.

### 4.8. Quantification of Total RNA

For planktonic and biofilm samples from day 7 containing a fixed number of total cells (4 × 10^7^), RNA extraction was performed according to the High Pure RNA Isolation Kit^TM^ protocol (Roche Diagnostics GmbH, Mannheim, Germany). The concentration of RNA solution was then determined using the NanoDrop Spectrophotometer, and the final values were presented as specific total RNA concentration (fg·cell^−1^). The analyzed samples originated from two independent experiments.

### 4.9. Quantification of eGFP Gene Transcription

The total RNA was first denatured by heating at 65 °C for 15 min, followed by rapid chilling on ice. The cDNA synthesis was carried out using the First Strand cDNA Synthesis Kit (Roche Diagnostics GmbH, Mannheim, Germany) according to the manufacturer’s recommendations. Briefly, each reaction contained Reaction Buffer (1× final concentration), MgCl_2_ solution (5 mM final concentration), dNTPs (1 mM final concentration), reverse primer (TGCCGGTGGTGCAGATGAAC), RNase inhibitor (50 units final concentration), AMV reverse transcriptase (≥20 units final concentration), gelatin (0.01 mg/mL final concentration), the total RNA sample and PCR grade water to complete 30 µL of reaction volume. The mixture was incubated at 25 °C for 10 min and at 42 °C for an additional 2 h. Then, the AMV reverse transcriptase was denatured by incubating the reaction at 99 °C for 5 min and cooling to 4 °C for a further 5 min. The relative amount of cDNA (and consequently of eGFP mRNA) was determined by RT-PCR, as previously described for PCN quantification, taking into account that the C_T_ values obtained are inversely proportional to the amount of eGFP cDNA in the sample. The final values per cell were presented in arbitrary units (A.U. cell^−1^) for comparative purposes.

### 4.10. Statistical Analysis

The results presented in Figure 1 and Figure 2 are an average of at least three independent experiments for each condition.

Average standard deviations (SDs) were calculated for all planktonic and biofilm parameters presented in Figure 1 and Figure 2. For biofilm formation (Figure 1), the following averages were obtained: SD < 2% for planktonic and biofilm total cells, SD < 3% for planktonic and biofilm viable cells, and SD < 7% for biofilm thickness. Concerning eGFP quantitation (Figure 2A)), SD < 16% and SD < 7% were obtained for planktonic and biofilm cells, respectively. Regarding plasmid copy number (Figure 2B), SD < 33% and SD < 40% were obtained for planktonic and biofilm cells, respectively. For eGFP mRNA and total RNA determination (Figure 3), SD < 8% and SD < 22% were obtained for planktonic and biofilm cells, respectively.

The data for biofilm formation (Figure 1) and analysis of transcription and total RNA (Figure 3) were analyzed using Statgraphics v6.0 software (Manugistics, Rockville, USA). Paired *t*-test analysis was performed for confidence levels of 90% (*p* values < 0.1; 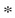) and 95% (*p* values < 0.05; 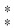 or 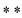). Paired *t*-test analysis (*p* values < 0.05) was also performed using Statgraphics software to compare biofilm and planktonic curves in Figure 2 (statistically significant differences between biofilm conditions were marked with †, while differences between planktonic conditions were indicated with ‡).

## 5. Conclusions

This work presents a contribution to the study of IPTG induction on heterologous protein expression in planktonic and sessile cells. To the best of our knowledge, this is the first report that presents a complete characterization of induction effects in both types of *E. coli* cells, including experimental data on plasmid stability, recombinant protein transcription, translation and cellular metabolic activity. This full molecular analysis was complemented with the theoretical determination of IPTG levels in suspension and on the liquid/biofilm interface. It is expected that the obtained results will be of great value to elucidate the mechanisms of induction on heterologous protein production, especially in biofilm cells, which have already shown potential to be used as protein factories.

## Figures and Tables

**Table 1:** Extracellular polymeric substance (EPS) analysis of induced and non-induced biofilms after 7 days of growth.

Biofilm Characteristics	Induced	Non-Induced
Biofilm mass (mg_biofilm_ cm^−2^)	4.00 ± 1.17	4.50 ± 0.870
Log cellular density (cells_·_cm^−2^)	7.83 ± 0.460	7.76 ± 0.460
Matrix proteins (mg g^−1^_biofilm_)	8.60 ± 1.80	9.40 ± 1.70
Matrix polysaccharides (mg g^−1^_biofilm_)	249 ± 41.0	231 ± 35.0

## Supplementary Materials

The following are available online at https://www.mdpi.com/1422-0067/21/2/576/s1, Figure S1: Time-course evolution of IPTG concentration within the flow cell system and eGFP production in planktonic and biofilm cells.

## Abbreviations

A	Area of the coupon (L^2^)
C	Bulk IPTG concentration (M L^−3^)
d	Hydraulic diameter (L)
D	Dilution rate (T^−1^)
$D_{aq}$	Diffusion coefficient of IPTG in water (L^2^ T^−1^)
F	Volumetric flow rate (L^3^ T^−1^)
$K_{m}$	External mass transfer coefficient (L T^−1^)
$\dot{n}$	Mass transfer rate (M T^−1^)
Re	Reynolds number (-)
Sc	Schmidt number (-)
Sh	Sherwood number (-)
t	Time (T)
V	Total system volume (L^3^)
τ	Average residence time (T)
