# Supplementary material for: The Impact of IPTG Induction on Plasmid Stability and Heterologous Protein Expression by Escherichia coli Biofilms
Source: Int J Mol Sci. 2020 Jan 16;21(2):576. doi: 10.3390/ijms21020576 (PMC7013871; doi:10.3390/ijms21020576)
Supplement: Supplementary file 1 [file ijms-21-00576-s001.pdf]

Supplementary material

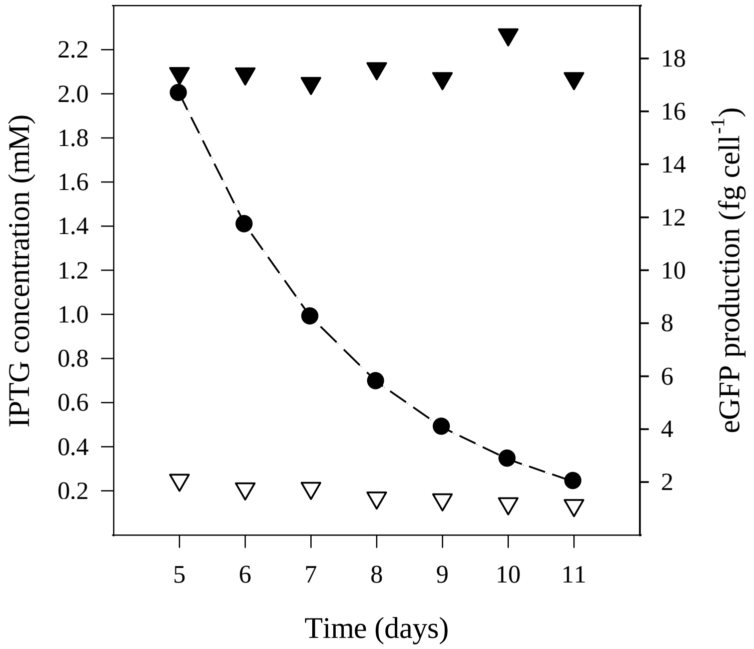

Figure S1. Time-course evolution of IPTG concentration within the flow cell system (‒●‒) (calculated from Equation (3)) and eGFP production in planktonic (▽) and biofilm (▼) cells. The culture was induced with 2 mM IPTG on day 5.
